# The hierarchical stratum response system of organism to microgravity during spaceflight

**DOI:** 10.1016/j.mmr.2026.100027

**Published:** 2026-04-27

**Authors:** Wei-Jia Sun, Rui-Kai Du, Yu-Heng Li, Guo-Hui Zhong, Jian-Wei Li, Zi-Zhong Liu, Xin-Xin Yuan, Xiao-Yan Jin, Shu-Kuan Ling, Ying-Xian Li

**Affiliations:** a National Key Laboratory of Space Medicine, China Astronaut Research and Training Center, Beijing 100094, China; b School of Medical Technology, Beijing Institute of Technology, Beijing 100081, China; c Oujiang Laboratory (Zhejiang Lab for Regenerative Medicine, Vision and Brain Health), Wenzhou Medical University, Wenzhou 325603, Zhejiang, China

**Keywords:** Spaceflight, Microgravity, Hierarchical stratum response system, Mechanical sensing

## Abstract

With the advancement of manned spaceflight technology, significant progress has been made in the fields of aerospace medicine and space biology to address the needs of astronauts in terms of health, safety, and mission performance. The physiological and pathological changes during microgravity exposure, including perception, response, and adaptation mechanisms, are gradually being unraveled. This study aimed to systematically summarize the common features of various molecules and structures within the cell membrane, cytoplasm, and nucleus responsible for sensing microgravity. It further examined the heterogeneous responses across different tissues, organs, and cells, as well as systemic-level interactions and synergies, and proposed a novel multilevel gravity-sensing theory. Additionally, the study highlighted cutting-edge in-orbit health monitoring technologies, including rapid fluid analysis using sensitive biomarkers and non-invasive evaluation using two-photon fluorescence microscopy. Furthermore, it explored the promising applications of emerging therapeutic approaches for microgravity adaptation, including small nucleic acid drugs targeting gene regulation, exosome-based drug delivery systems, and natural small-molecule drugs aimed at combating microgravity effects.

## Background

Microgravity is the most distinct environmental factor during spaceflight that differs from terrestrial conditions. It is a mechanical environment in which an object exhibits near-zero gravity in a state of free fall. The most typical scenario of microgravity is produced by a spacecraft in orbit moving around the Earth [1]. Life on Earth has adapted to the 1 g gravitational environment through long-term evolutionary processes. Once in orbit, where organisms experience microgravity, the entire physiological system exhibits temporal, systemic adaptive alterations involving multiple organ systems [2]. The changes of the organism during microgravity are exhibited at the molecular, cellular, tissue, and organ levels, forming the multi-tiered response system within the body.

**Fig. 1:**
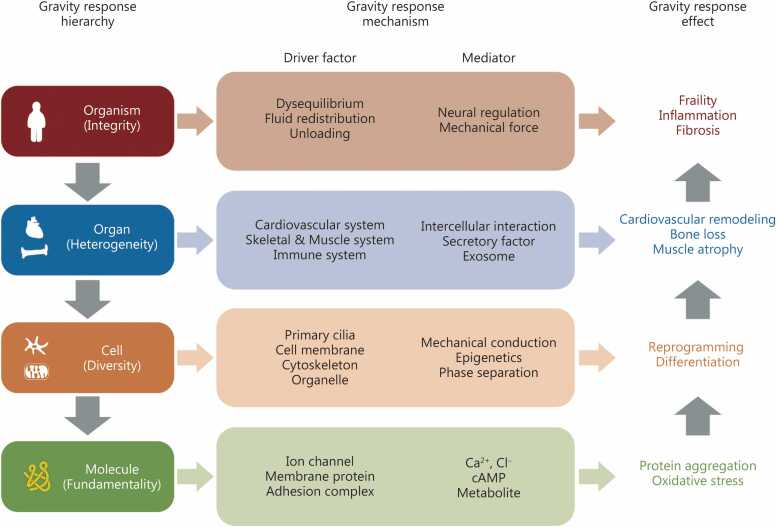
**Multi-level response of an organism to gravity.** In microgravity conditions, the entire organism exhibits temporally sequential, systemic, and adaptive characteristics across different tissues and organs. The gravitational responses spanning molecular, cellular, tissue, and organ levels constitute the organism’s multi-level response system. cAMP. Cyclic adenosine.

Structures like the extracellular matrix, cell membrane, and organelles can detect gravitational changes, with ion channels acting as key sensors [3]. Intracellular calcium signaling mediates a series of downstream pathways [4]. Moreover, epigenetic and post-translational modifications (PTMs) provide dynamic precision regulation [5]. At the cellular level, responses are heterogeneous across cell types, including stem cell differentiation, proliferation, apoptosis, and tissue remodeling [6], [7]. At the tissue and organ level, prolonged microgravity exposure induces aging-like symptoms across multiple systems, including bone loss, cardiovascular remodeling, immune suppression, and metabolic disorders [8]. Systemic organ interaction is mediated through secreted factors, exosomes, hormones, or neurotransmitters, etc., which coordinate whole-body adaptation and homeostasis under gravitational stress [9], [10] (Fig. 1).

Based on these fundamental research findings, it is urgent to develop real-time, rapid, sensitive, and reliable monitoring and early warning technologies, while simultaneously gaining in-depth insights into the body’s physiological responses to gravitational changes as human deep space exploration advances. Concurrently, there is an urgent need to develop more precise and effective protective countermeasures.

This review systematically integrates recent advances in microgravity biology, space medicine, and on-orbit health protection technologies. It elucidates the biological response mechanisms to gravitational changes from the molecular to the systemic level, providing scientific foundations and strategic support for mitigating health risk and advancing technologies essential to development in long-term crewed deep space missions.

## Cell structure and related molecules sensing gravity changes

**Fig. 2:**
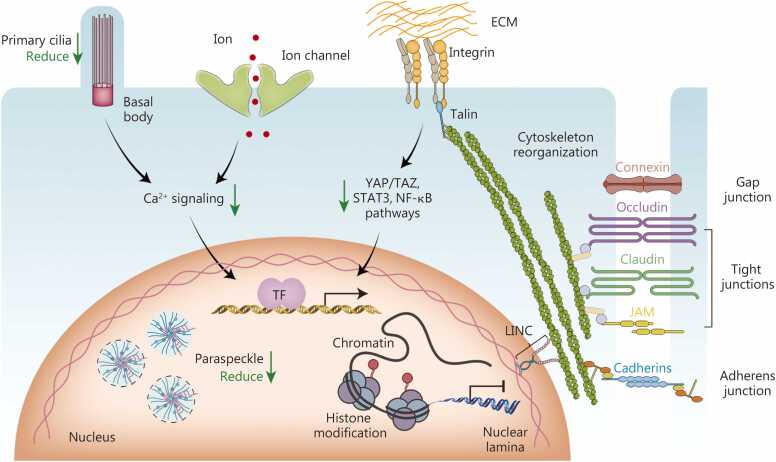
**The response mechanism of the cell to microgravity.** In microgravity conditions, alterations occur in various subcellular structures within cells. The cytoskeletal system, cell membrane, specialized nuclear structures, primary cilia, and extracellular matrix (ECM) all perceive the absence of gravity and undergo modifications, consequently affecting cellular functions and organismal adaptation processes. YAP. Yes-associated protein; TAZ. Transcriptional co-activator with PDZ-binding motif; STAT. Signal transducer and activator of transcription; NF-κB. Nuclear factor κB; LINC. Linker of nucleoskeleton and cytoskeleton; JAM. Junctional adhesion molecule.

Cells respond to gravitational changes through multiple structural alterations, including changes in the cell membrane and membrane proteins, cytoskeletal polymerization, the structure of chromosomes and membrane-less organelles in the nucleus, and extracellular matrix (ECM)- mediated cellular signaling transduction and cell-cell interactions, thereby influencing cellular functions under microgravity (Fig. 2).

### Cell membranes and membrane proteins

The cell membrane serves as a barrier separating the internal and external environments, where membrane integrity is crucial for barrier function [11]. Studies have demonstrated that the fluidity of lipid membranes increases significantly under reduced gravity, manifested as decreased viscosity [12], [13], thereby both facilitating the repair of membrane defects and directly influencing the dynamics of ion channels [14]. Consequently, variations in gravity can regulate specific ion channel functions.

Mechanosensitive ion channels are unique mechanosensory membrane proteins that convert mechanical stimuli into electrical/chemical signals [15]. Piezo type mechanosensitive ion channel component 1 (PIEZO1) is a mammalian mechanosensitive cation channel [16], [17]. Under membrane tension, it flattens and undergoes conformational changes (blade expansion, cap rotation) to open its pore. Localized in membrane invaginations, it senses force, interacts with the cytoskeleton, and converts mechanical force into Ca²⁺ currents [18]. In the skeletal system, the absence of mechanical forces and microgravity reduced osteoblast function by suppressing PIEZO1 expression and activity [19], [20]. The transient receptor potential (TRP) multigene superfamily encodes another important class of mechanosensory members. Transient receptor potential vanilloid 4 (TRPV4) can sense mechanical forces in chondrocytes [21], osteocytes [22], epithelial cells [23], and endothelial cells [24]. Microgravity reduces TRPV4 activity, thereby inhibiting the normal function of osteoblasts and chondrocytes [25]. Moreover, the downregulation of the transient receptor potential canonical (TRPC) channel plays a significant role in unload-induced muscle atrophy [26].

A study has highlighted the crucial role of anion channels in gravity sensing [27]. The calcium-activated chloride channel (Ano1) regulates osteoclast differentiation and function by influencing extracellular acidification and receptor activator of nuclear factor κB (NF-κB) ligand signaling pathway [28]. Ano1 shares structural and evolutionary similarities with the typical mechanosensitive ion channel hyperosmolarity-gate calcium-permeable channels [29]. Ano1 mediates osteoclast responses to gravity through calcium-dependent channel activity. Mechanical unloading enhances its activity, decreases intracellular chloride concentration, and activates calcium signaling [27]. These findings provide novel therapeutic targets for combating microgravity-induced bone loss and osteoporosis. Moreover, transmembrane proton flow mediated by hydrogen ion (H⁺) channels has been validated as an important gravitational signaling mechanism [30]. When subjected to gravitational stimulation, the meristematic regions of plants establish significant proton gradients through the polar distribution of plasma membrane H⁺-ATPase [30]. This phenomenon is especially pronounced in the root elongation zone, where active H⁺ efflux acidifies the cell wall to promote expansion and generates proton motive force driving ion transport by establishing transmembrane differences in electrochemical potential [31]. The conservation of these transmembrane electrophysiological characteristics proposed the hypothesis that homologous voltage gated proton channels participate in gravity-sensing processes through similar electrochemical kinetic mechanisms in animal cells.

### Cytoskeleton

The eukaryotic cytoskeleton, composed of microtubules, microfilaments, and intermediate filaments, maintains cell morphology and function [32]. External forces are transmitted to the actin cytoskeleton through adhesion complexes such as focal adhesions [33], [34]. The linker of nucleoskeleton and cytoskeleton (LINC) complex further links actin to nuclear lamina, potentially transmitting external forces to chromatin [35]. Furthermore, microgravity alters cytoskeletal structures, particularly microfilaments and associated proteins, which, in turn, interfere with signal transduction, cell growth, metabolic synthesis, and cytokine/hormone secretion, ultimately impacting cellular function [36], [37], [38], [39]. Microgravity reorganizes microtubules and F-actin, altering cell morphology, adhesion, and gene expression [40], [41]. It increases F-actin density but loosens stress fibers [40], a change observed in osteoblasts and monocytes as a reduction in stress fiber density [7], [42]. In addition, spaceflight can induce functional decline and microtubule depolymerization in cultured cardiomyocytes [43]. But thioredoxin protects the microfilament skeleton in cardiomyocytes exposed to simulated microgravity conditions by modulating redox status using rotating wall vessel culture [37], [44], [45], [46].

Primary cilia are microtubule-based organelles essential for cellular signaling and environmental sensing [47]. Under simulated microgravity, the length of primary cilia in osteoblasts is significantly shortened or even completely disappears, inhibiting osteoblast differentiation and reducing mineralization capacity [48]. And microgravity interferes with mechanotransduction by altering the activity of ion channels (e.g., TRPV4), the expression of transport proteins and receptor proteins on primary cilia, Ca²⁺ overload, oxidative stress, and suppressed osteogenesis [49], [50], [51]. However, the structure and function of primary cilia in microgravity and their precise regulatory roles remain poorly understood. Further investigation of these issues is crucial for understanding the mechanisms of cellular gravity sensing.

### Cell nucleus

The structures within the cell nucleus can sense the changes in gravity. The three-dimensional structure of chromosomes and the organization of chromatin undergo alterations under microgravity conditions. These changes may lead to modifications in gene expression patterns, subsequently influencing cellular function and adaptive capacity [52]. Moreover, microgravity may influence epigenetic modifications on chromosomes, such as DNA methylation and histone modifications [53]. Simultaneously, DNA damage repair disruption in spaceflight results from the combined effects of microgravity and cosmic radiation exposure, with radiation being the primary driver of DNA strand breaks while microgravity may impair cellular repair mechanisms, together leading to increased frequencies of chromosomal abnormalities and gene mutations [54]. This poses risks to astronauts’ health and to the safety of long-term space missions. In summary, the effects of microgravity on chromosomes are a complex research hotspot. Further investigations may help elucidate the adaptive mechanisms of chromosomes in microgravity, thereby providing key theoretical foundations for long-duration spaceflight and advancing space biology.

Membrane-less organelles, represented by paraspeckles, are another type of structure in the nucleus that can sense changes in gravity. Mechanical stimulation (including hypergravity, microgravity, fluid shear stress, and substrate stiffness) dynamically modulates the morphology and quantity of membrane-less organelles to regulate biological processes. Microgravity can reduce nuclear paraspeckle assembly transcript 1 (Neat1) expression (the core component of paraspeckles) and inhibit paraspeckle formation in osteoblasts, thereby impairing osteoblast differentiation and suppressing bone formation [55]. These findings reveal a novel mechanism by which microgravity regulates bone metabolism by affecting the Neat1/paraspeckle nuclear architecture.

### Cell-extracellular matrix and cell-cell contacts

The sensing of microgravity at cell-ECM and cell-cell contacts involves complex molecular and cellular mechanisms, with the cellular response primarily manifested as dynamic changes in ECM composition, regulation of cell-ECM and cell-cell interactions, and activation of mechanotransduction pathways [56], [57]. Microgravity disrupts the composition and structure of the ECM by modulating the expression of ECM-related genes and proteins [58]. In bone tissue, it leads to upregulated expression of matrix metalloproteinases (e.g., matrix metallopeptidase 13), disorganized collagen deposition, and accelerated ECM degradation [59]. Similar ECM remodeling occurs in skin and heart tissues, affecting tissue integrity and cardiac adaptation [60]. These changes are closely linked to impaired cell-ECM adhesion and disrupted mechanotransduction. Microgravity alters the mechanical properties of ECM, for example, by reducing tissue stiffness, thereby modulating intracellular signaling pathways, including calcium signaling and Rho GTPases, and affecting cell proliferation, differentiation, and apoptosis [57], [61]. In cartilage and bone tissues, these mechanical cues activate inflammatory signaling [e.g., signal transducer and activator of transcription 3 (STAT3) and NF-κB], leading to the upregulation of interleukin-6 (IL-6) and MMP3, ultimately promoting tissue degeneration [62]. Additionally, microgravity alters the localization and activity of focal adhesion proteins [e.g., integrin, focal adhesion kinase, paxillin, and vinculin] and diminishes physical contact between cells and the ECM. This disruption inhibits integrin-mediated focal adhesion signaling, resulting in cytoskeletal disorganization, nuclear envelope dysfunction (e.g., LINC complex dysfunction), and transcriptional reprogramming [e.g., suppression of yes-associated protein/transcriptional co-activator with PDZ-binding motif (YAP/TAZ) and runt-related transcription factor 2 (RUNX2)] [63]. Microgravity also impairs focal adhesion dynamics [e.g., the IQ motif-containing GTPase-activating protein 1/HCLS1-associated protein X-1 (IQGAP1/Hax1) interaction], further inhibiting cell migration [64]. Collectively, these findings highlight the ECM as a gravity-sensitive interface through which microgravity reshapes cell behavior and tissue function. Furthermore, microgravity compromises tissue barrier integrity, intercellular communication, and mechanical signaling by modulating the expression, distribution, and stability of intercellular junction proteins, thereby eliciting a range of physiological adaptive responses. For instance, exposure to microgravity downregulates tight junction proteins (such as occludin, claudin, and zona occludens protein 1) and vascular endothelial-cadherin in vascular endothelial cells, resulting in the loss of junctional structures, weakened intercellular adhesion, and aberrant endothelial cell migration and angiogenesis [65], [66], [67].

In summary, the coordination unity between the intracellular and extracellular environments is crucial for normal cellular function. Cells adapt to microgravity through internal self-regulation and interactions with their surroundings. The changes in molecular structures and their systemic impacts on cellular function under microgravity constitute a complex yet critical research domain. Understanding these microgravity-induced cellular adaptations is essential for elucidating life’s resilience in altered gravity environments. Future studies should unravel communication mechanisms between intracellular structures and the coordination between cells under microgravity, thereby providing valuable insights for human space exploration and life sciences.

## Calcium signaling mechanism underlying gravity transduction

### Effects of microgravity on calcium homeostasis

**Fig. 3:**
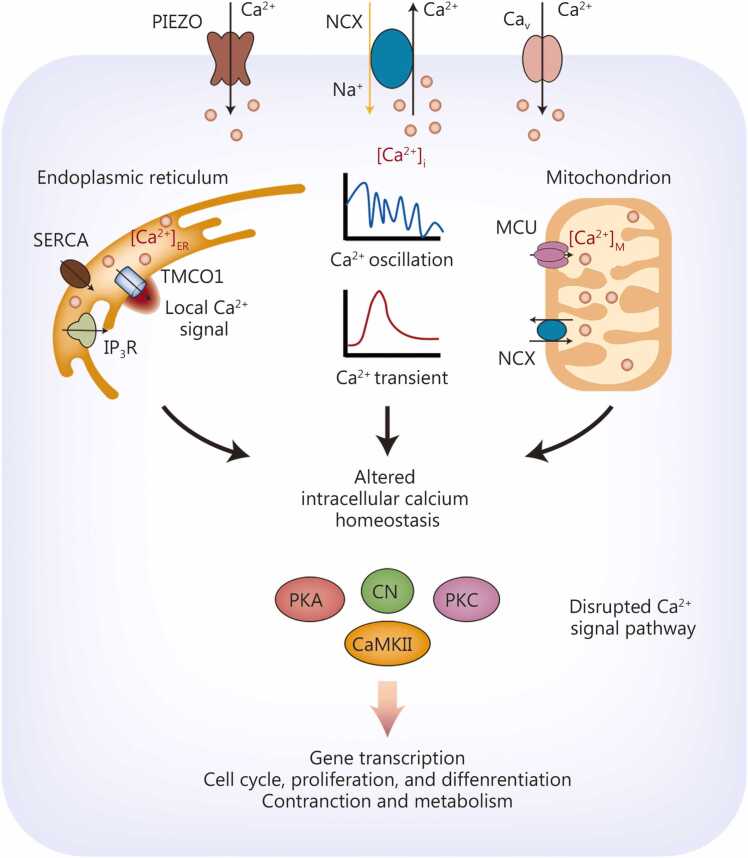
**Calcium signal mediates gravity changes.** When the gravitational environment changes, cells can perceive this alteration, subsequently triggering spatiotemporal dynamics of free calcium ion concentration (Ca^2+^) across multiple scales, hierarchical levels, and orders of magnitude in both cytoplasm and localized microdomains partitioned by organelles such as endoplasmic reticulum and mitochondria. NCX*.* Na⁺/Ca²⁺ exchanger; SERCA. Sarco/endoplasmic reticulum Ca²⁺-ATPase; TMCO1. Transmembrane and coiled-coil domains 1; MCU. Mitochondrial calcium uniporter; PKA. Protein kinase A; CN. Calcineurin; CaMKII. Ca²⁺/calmodulin-dependent protein kinase II; PIEZO. Piezo type mechanosensitive ion channel component; IP_3_R. Inositol 1,4,5-trisphosphate receptor; [Ca^2+^]_ER_. Calcium concentration in the endoplasmic reticulum; [Ca^2+^]_i_. Intracellular free calcium ion concentration; [Ca^2+^]_M_. Mitochondrial calcium ion concentration; PKC. Protein kinase C.

In the environment of spaceflight, cells can perceive gravitational variations, which induce spatiotemporal dynamics in free Ca^2+^ concentration across multiple scales, levels, and orders of magnitude in extracellular spaces, cytoplasm, and organelle-partitioned microdomains, and significant alterations occur in calcium homeostasis and intracellular calcium signaling pathways in the body, which play a central role in multisystem adaptive regulation [68], [69]. At the whole-organism level, astronauts in space experience pronounced disturbances in calcium metabolism, as reflected by elevated serum and urinary calcium levels and reduced intestinal calcium absorption [70]. In bone, osteoblasts respond to unloading with reduced intracellular Ca²⁺ and bone formation [71], whereas osteoclasts exhibit elevated intracellular Ca²⁺ and amplified oscillations that accelerate differentiation and bone resorption [72]. Enhanced bone resorption leading to dissolution of bone matrix minerals, releasing large amounts of free calcium that subsequently disrupts systemic calcium homeostasis [73]. Modulating calcium-phosphorus metabolism can partially mitigate spaceflight-induced bone loss [74]. However, the complexity of the problem lies in the fact that the effects of microgravity or simulated microgravity extend beyond bone. It causes significant alterations in intracellular calcium concentrations and disrupts normal calcium signaling in various cells, including cardiomyocytes [75] and skeletal muscle cells [76]. Ca²⁺ responses are tissue-specific; for example, simulated microgravity dampens cytosolic Ca²⁺ in lymphocytes, while simultaneously elevating the signal in germ and smooth-muscle cells [77], [78], [79]. Thus, the same gravitational cue is translated into distinct Ca²⁺ dialects that orchestrate organ-specific adaptation. The generation and propagation of abnormal intracellular calcium signals represent a common molecular mechanism by which different tissues respond to changes in gravity (Fig. 3).

### Ca²⁺ downstream signaling mediates the body’s response to gravity changes

Spaceflight-induced calcium metabolism disorders affect calcium concentration levels and their dynamic patterns (e.g., calcium transients, oscillations, and waves) through plasma membrane calcium channels and transporter-mediated calcium transport processes across various tissue cells [80], [81]. These alterations subsequently regulate cellular functions via downstream calcium signaling pathways such as Ca²⁺/calmodulin (CaM)-dependent protein kinase II (CaMKII) and calcineurin [82], [83]. Li *et al.* [65] first demonstrated that cardiomyocytes respond to gravitational changes, specifically microgravity, which significantly increases cytosolic calcium concentration and oscillation frequency, thereby activating the downstream p-CaMKII/histone deacetylase 4 (HDAC4) signaling pathway to promote cardiomyocyte hypertrophy. These cytosolic calcium dynamics were later confirmed in human induced pluripotent stem cell (iPSC)-derived cardiomyocytes and neonatal cardiovascular progenitor cells during actual spaceflight [84], [85]. Both real and simulated microgravity significantly activate calcium-dependent signaling pathways crucial for myocyte contraction and function [86], [87]. The Ca²⁺/CaMKII signaling pathway is known to be a crucial regulator of osteoblast behavior [20]. The chronic reduction in Ca²⁺ influx under microgravity would lead to diminished CaMKII activity, crippling downstream signaling events required for osteoblast maturation and function [20]. Calcineurin is a Ca²⁺-dependent, CaM-stimulated protein phosphatase. By dephosphorylating target proteins, including transcription factors, calcineurin plays a role in modulating bone mineralization [88]. A deficit in Ca²⁺ signaling would logically lead to reduced calcineurin activity, further contributing to the impaired ability of osteoblasts to form new mineralized matrix.

### Endoplasmic reticulum calcium-mediated gravitational signal transduction

The endoplasmic reticulum (ER) is the primary intracellular calcium store and is central to calcium signaling [89]. ER calcium channel-mediated calcium dynamics are also critical for regulating cellular responses to external stimuli [90]. These external stimuli can induce ER calcium overload, thereby triggering ER stress and subsequent physiological/pathological changes. Conversely, inhibiting ER-mediated calcium signaling also leads to cellular dysfunction [91], [92], [93], [94]. Simulated microgravity/hypergravity significantly alters resting calcium levels and calcium transients within the ER, thereby activating downstream calcium-dependent pathways [75]. Further investigations revealed that TMCO1, a calcium channel activated by ER calcium overload, shows significantly reduced expression in osteoblasts under simulated microgravity [94]. TMCO1 deficiency increases ER calcium concentration while attenuating localized microdomain calcium signaling near the ER membrane, suppressing downstream CaMKII pathway activation and ultimately contributing to bone loss [94]. Notably, TMCO1-deficient osteoblasts exhibit reduced responsiveness to gravitational stimuli [94]. Sarco/endoplasmic-reticulum Ca²⁺-ATPase (SERCA) serves as the principal pump that ferries cytosolic Ca²⁺ back into the ER [95]. Post-flight mice exhibited significantly elevated expression of the ER calcium pump SERCA in soleus and tibialis anterior muscles, accompanied by altered changes in calcium uptake capacity and diminished muscle contractility [96]. Moreover, the decline in smooth muscle cell contractility correlated with reduced expression of the ER calcium channel ryanodine receptor (RyR) [87], [96]. Under tail-suspension simulated microgravity conditions, RyR2-mediated ER calcium release frequency increases substantially in rat and mouse cardiomyocytes, leading to cardiac dysfunction [97], [98]. Considering the essential role of ER-mediated calcium homeostasis and signaling in tissue/organ development and function, further elucidation of the regulatory mechanisms governing ER calcium channel-mediated calcium signaling under gravitational changes is imperative.

### Microgravity-induced mitochondrial dysfunction through calcium metabolism imbalance

Mitochondria, the cell’s energy producers, rely on calcium ions to regulate adenosine triphosphate (ATP) production[99]. However, excessive calcium uptake during stress leads to overload, triggering harmful reactive oxygen species (ROS) production and cell death [100]. Conversely, blocking mitochondrial calcium uptake reduces ATP production and activates AMP-activated protein kinase (AMPK) [101]. Activated AMPK induces autophagy by inhibiting mammalian target of rapamycin, which plays a pro-survival role under this energy stress condition [101]. Therefore, maintaining mitochondrial calcium homeostasis is extremely important for the normal functioning of cells. Recently, the in-depth analysis of the biomedical data of 59 astronauts from the International Space Station (ISS) and hundreds of biological samples preserved in the NASA GeneLab, including multi-omics and systems biology studies at the transcriptomics, proteomics, metabolomics, and epigenetics levels, has revealed that mitochondrial dysfunction is a common characteristic of various tissues and organs of the body under spaceflight conditions [53]. Further, mitochondrial calcium concentrations, a key regulated parameter in intracellular calcium trafficking, are directly influenced by cytoplasmic and ER calcium homeostasis [102]. Consequently, a disruption of intracellular calcium balance inevitably impairs mitochondrial calcium regulation, compromising its essential functions [103]. Simulated microgravity can significantly reduce the calcium ion concentrations in the mitochondria of vascular smooth muscle cells, thereby inhibiting mitochondrial function [77]. These findings suggest that the calcium imbalance in mitochondria caused by microgravity may be an important reason for mitochondrial dysfunction. Therefore, it can be presumed that the mitochondrial calcium metabolic imbalance and calcium-related signal alterations induced by gravity changes are the common mechanisms by which cells respond to microgravity. These disturbances are also key contributors to mitochondrial dysfunction in various tissues and cells of the body.

## Accurate regulation of microgravity signals

The sensing and transduction of mechanical signals by cells is a precisely regulated process in microgravity. Cells perceive the changes in the intensity of extracellular mechanical forces and transmit these signals into the cells. These signals then influence processes such as gene transcription, translation, and PTMs through signaling pathways, thereby precisely regulating cellular functions [104].

### Epigenetics

#### DNA methylation

DNA methylation patterns may undergo alterations under microgravity, influencing gene expression. The expression of both DNA methyltransferase 1 (*DNMT1*) and *HDAC1* genes was found to be downregulated in human T lymphocytes [105]. Reduced expression of the histone methyltransferase SET domain bifurcated histone lysine methyltransferase 1 (Setdb1) in osteoblasts leads to the upregulation of miR-212-3p, which in turn downregulates high mobility group box 1 and consequently inhibits osteogenic differentiation [106]. Meanwhile, Setdb1 catalyzed the formation of histone H3 lysine 9 trimethylation (H3K9me3) in the promoter region to suppress the expression of macro domain containing 2; this expression was upregulated and subsequently inhibited osteoblast proliferation through the glycogen synthase kinase-3β/β-catenin signaling pathway [107]. Bone marrow mesenchymal stem cells (BMSCs) exhibit aberrant expression of histone demethylase phd finger protein 8, which suppresses osteogenic differentiation by modulating the promoter methylation levels of osteogenesis-related transcription factors such as RUNX2 [108].

#### Histone modification

The intracellular regulatory mechanisms of histone modifications under microgravity involve dynamic equilibrium and metabolic interactions of multiple epigenetic modifications. Microgravity disrupts the modification patterns of H3K4me3 and H3K27me3 in embryos, impairing zygotic gene activation and consequently affecting developmental processes [109]. The activation of the NAD^+^-dependent deacetylase silent information regulator 3 mitigates microgravity-induced muscle cell death while maintaining the expression of differentiation markers [110]. The trimethylation of H3K27me3 exhibits significant alterations under microgravity conditions [111], [112]. In Caenorhabditis elegans (*C. elegans*), microgravity raises H3K27me3 across the domain of unknown function 19 (*DUF-19*) gene cluster, whereas this modification is markedly reduced in *HDAC4* mutants, indicating that HDAC4 is required for the H3K27me3 response. Furthermore, microgravity activates HDAC4 and suppresses the neural differentiation-related genes [e.g., T20D4.11-like domain-containing protein (*T20D4.11*)], thereby influencing the growth and development of *C. elegans* [111]*.* Simulated microgravity inhibits neural differentiation-related histone modifications, such as H3K27me3 enrichment, in mesenchymal stem cells through cytoskeleton-nuclear lamina structural reorganization, diminishing cell differentiation capacity [112]. In summary, microgravity modulates gene expression by altering the balance of histone methylation/acetylation, in coordination with epigenetic mechanisms such as cellular structural remodeling and DNA methylation. These changes ultimately influence cell differentiation, metabolism, and systemic adaptability.

#### Noncoding RNA

Noncoding RNAs are transcriptional products of the genome playing key regulatory roles in physiological and pathological processes such as stem cell maintenance, cell differentiation, apoptosis, metabolism, and signal transduction [113], [114]. These noncoding RNAs mainly include microRNAs (miRNAs), long noncoding RNAs (lncRNAs), and special noncoding RNAs derived from the 3’-untranslated region (3’-UTR) (Table 1) [55], [115], [116], [117], [118], [119], [120], [121], [122], [123], [124], [125], [126], [127], [128], [129], [130], [131], [132].

**Table 1:** Precise regulation of noncoding RNA in response to weightlessness.

**Changes in noncoding RNA**	**Cells**	**Target**	**Biological process**	**References**
miRNA				
miR-214↑	Osteoblast Osteoclast	*Atf4* *Pten*	Inhibit osteoblast function Promote osteoclast activity	[115], [116]
miRNA-129-3p↓	Osteoblast	*BMP2*	Cilia loss and impaired osteoblast function	[117]
miR-181c-5p↑	Osteoblast	*Cyclin B3*	Inhibit osteoblast function	[118]
miRNA-103↑	Osteoblast	*Cav1.2*	Inhibit osteoblast proliferation	[119]
miRNA-132-3p↑	Osteoblast	*Ep300*	Inhibit osteoblast differentiation	[120]
miR-494↑	Osteoblast	*BMP*	Inhibit osteoblast differentiation	[121]
miR-503-5p↑	HPMECs	*Bcl-2*	Promote cell apoptosis	[122]
miR-27b-5p↓	HUVECs	*ZHX1*	Promote cell apoptosis	[123]
Let-7↓	*C. elegans*	*SKN-1*, *Nrf*	Protect sports ability	[124]
miR-223↑	Liver	*CDK2*, *CUL1*	Inhibit hepatocyte proliferation	[125]
miR-21↓	T-cell	*BTG2*, *TAGAP, SPRY2*, *FASLG*	Inhibit T cell activation	[126]
LncRNA				
Neat1↓	Osteoblast	*Smurf1*	Inhibit osteoblast function	[55]
HOTAIR↓	Osteoblast BMSC	miR-214 *Runx2*, *Sp7*	Inhibit osteoblast function	[130]
ODSM↓	Osteoblast	miR-1-139p/*ELK3*	Inhibit osteoblast differentiation	[129]
MALAT1↓	Osteoblast	miR-217/*AKT3*, miR-485-5p/*WNT7B*	Inhibit osteoblast differentiation	[127], [128]
MUMA↓	Myocyte	miR-762/*MyoD*	Reduced muscle production	[131]
CKIP-1 3’UTR↑	Cadiomyocyte	Let-7f/*CaMKK2*	Inhibit myocardial remodeling	[132]

↑indicates an increase under microgravity; ↓indicates a decrease under microgravity. Atf4. Activating transcription factor 4; Pten. Phosphatase and tensin homolog; BMP. Bone morphogenetic protein; Cav1.2. L-type voltage gated calcium channel 1.2; Ep300. E1A binding protein p300; Bcl-2. B-cell lymphoma 2; ZHX1. Zinc fingers and homeoboxes 1; SKN-1. SKiNhead1; Nrf. Nuclear factor erythroid 2-related factor; CDK2. Cyclin-dependent kinase 2; CUL1. Cullin-1; BTG2. BTG family, member 2; TAGAP. T-cell activation; SPRY2. Sprouty homolog 2; FASLG. Fas ligand; Smurf1. SMAD-specific E3 ubiquitin protein ligase 1; Runx2. Runt-related transcription factor 2; ELK3. ETS-domain protein; AKT3. Akt serine/threonine kinase 3; WNT7B. Wnt family member 7B; MyoD. Myogenic differentiation; Neat1. Nuclear paraspeckle assembly transcript 1; Sp7. Sp7 transcription factor; CaMKK2 Ca²⁺/calmodulin-dependent protein kinase kinase 2; *C. elegans.* Caenorhabditis elegans

Aberrant expression of noncoding RNAs under microgravity is closely associated with key physiological processes, including microgravity-induced bone loss, vascular remodeling, liver dysfunction, and systemic immune suppression. MiR-214 suppresses osteoblast activity by targeting the activating transcription factor 4 (*Atf4*) [115], while simultaneously promoting osteoclast function through phosphatase and tension homolog inhibition [116]. Thus, during microgravity-induced bone loss, elevated miR-214 contributes to bone loss by reducing bone formation and enhancing bone resorption, suggesting miR-214 as an important diagnostic biomarker, and antisense oligonucleotides targeting miR-214 significantly inhibit bone loss [115], thereby offering a novel therapeutic strategy for spaceflight-associated bone loss. Many other miRNAs participate in the functional regulation of osteoblasts under simulated microgravity. For instance, the levels of miRNA-129-3p are markedly reduced in osteoblasts, leading to altered primary cilium length. Supplementation of this miRNA alleviates microgravity-induced cilium loss and osteoblast dysfunction [117]. Elevated expression of specific miRNAs inhibits the differentiation and function of osteoblasts. Among these, miR-181c-5p impairs osteoblast function by targeting cyclin B3 [118]. MiR-103 suppresses osteoblast proliferation by inhibiting L-type voltage gated calcium channel 1.2 (*Cav1.2*) expression [119]. Both miRNA-132-3p and miR-494 inhibit osteoblast differentiation; miRNA-132-3p acts by targeting the transcriptional co-activator E1A binding protein p300 (*Ep300*) [120], while miR-494 functions by attenuating bone morphogenetic protein (BMP) signaling [121].

Vascular endothelial cells also exhibit differential miRNA expression under simulated microgravity. Eight differentially expressed miRNAs are identified in pulmonary microvascular endothelial cells, among which miR-503-5p promotes apoptosis by targeting B-cell lymphoma 2 (*Bcl-2*) [122]. In human umbilical vein endothelial cells (HUVECs), miR-27b-5p influences apoptosis via zinc fingers and homeobox 1 (*ZHX1*) [123]. In *C. elegans,* the dysregulated expression of 19 miRNAs is associated with suppressed motor behavior and increased toxicity under microgravity [124]. Similarly, in rat liver, upregulated miR-223 inhibits hepatocyte proliferation [125]. Notably, in T cells cultured aboard the ISS, reduced miR-21 levels correlate with impaired T cell activation, indicating that gravity regulates T cell function through noncoding RNA mechanisms [126].

LncRNAs represent another class of molecules that exert precise regulatory functions in the physiological effects of weightlessness. Some lncRNAs are significantly downregulated under simulated microgravity conditions to facilitate osteogenic differentiation. Among them, lncRNA MALAT1 sponges miR-217 or miR-485-5p [127], [128], and lncRNA ODSM inhibits osteoblast apoptosis and promotes cell differentiation [129], and Neat1 forms a phase-separated structure called paraspeckle that sequesters SMAD-specific E3 ubiquitin protein ligase 1 (*Smurf1*) mRNA [55]. Meanwhile, another lncRNA, HOTAIR, responds to gravitational changes through nuclear translocation, thereby regulating the osteogenic differentiation process of BMSCs [130]. LncRNA MUMA was downregulated in muscle atrophy, impairing myogenesis, which can sponge miR-762 to enhance myoblast differentiation [131].

Furthermore, the noncoding sequences of coding RNAs have emerged as a novel class of regulatory molecules, playing crucial roles in various biological processes. A special noncoding RNA derived from the 3’-UTR of casein kinase 2 interacting protein-1 (CKIP-1), existing independently of *CKIP-1* mRNA, has been identified [132]. The *CKIP-1* 3’-UTR exerts its protective effects by activating the AMPK-peroxisome proliferator-activated receptor α-carnitine palmitoyltransferase 1b axis to enhance cardiac fatty acid metabolism, thereby counteracting cardiac remodeling induced by simulated microgravity [132].

### Protein post-translational modifications

**Fig. 4:**
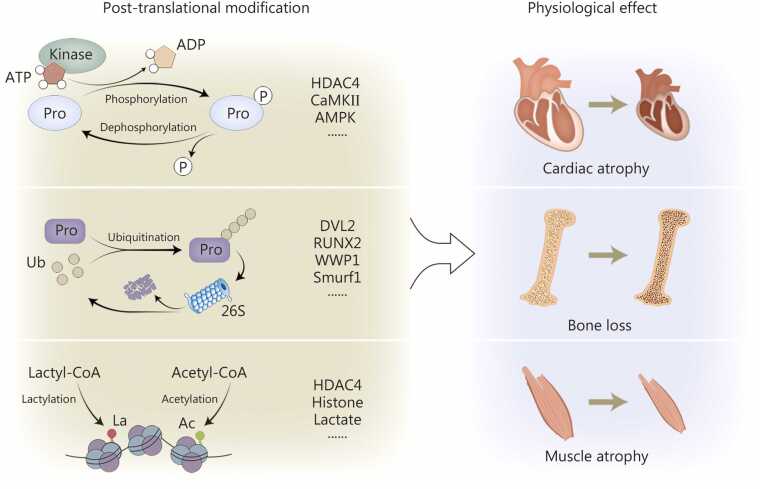
**The role of protein post-translational modification in the regulation of weightlessness signals.** In the microgravity environment of spaceflight, both extracellular and intracellular signaling undergo corresponding changes, wherein alterations in phosphorylation, ubiquitination, and lactylation play important regulatory roles in microgravity-induced myocardial remodeling, bone loss, and muscle atrophy. HDAC4. Histone deacetylase 4; CaMKII. Ca²⁺/calmodulin-dependent protein kinase II; AMPK. AMP-activated protein kinase; DVL2. Dishevelled segment polarity protein 2; RUNX2. Runt-related transcription factor 2; WWP1. WW domain-containing E3 ubiquitin protein ligase 1; Smurf1. Smad ubiquitination regulatory factor 1; Pro. Protein; Ub. Ubiquitination; La. lactylation; Ac. Acetylation.

PTMs lie at the core of many cellular signal transduction events [133]. Recent studies have revealed that weightlessness or simulated microgravity induces significant metabolic alterations in organisms, which further influence the regulation of PTMs [134], [135]. When stimulated by extracellular signals such as environmental forces and neuroendocrine factors, the proteins involved in signal transduction are regulated through PTMs, including phosphorylation, ubiquitination, lactylation, acetylation, and glycosylation [136]. These covalent modifications are transient and reversible, playing essential roles in protein function and signal transduction. Among these, phosphorylation, ubiquitination, and lactylation are common forms of protein modification in eukaryotic cells [137], [138], [139]. The biological signals they mediate are involved in regulating cellular proliferation, development, differentiation, apoptosis, and remodeling [137], [138], [139]. Besides individual conventional protein PTMs, these multiple protein PTMs also work in coordination, serving as a synergistic fine-tuning mechanism to precisely regulate the cellular response to even the slightest changes in the environment [140] (Fig. 4).

Protein phosphorylation and dephosphorylation are the most prevalent and crucial forms of PTMs in biology, with approximately one-third of cellular proteins estimated to undergo phosphorylation [141]. Numerous extracellular signals can induce protein phosphorylation in cells, thereby altering downstream signaling pathways and participating in vital biological processes such as gene expression and hormone secretion [142], [143]. A previous study showed that the phosphorylation level of RyR2, a crucial calcium channel protein in cardiomyocytes, was significantly elevated after 56 d of tail suspension in mice, leading to abnormal calcium signaling. This was considered a potential contributor to microgravity-induced cardiac arrhythmias [98]. Further research revealed that after 28 d of tail suspension, the phosphorylation levels of key proteins regulating pathological cardiac remodeling, HDAC4, extracellular regulated protein kinases (ERK1/2), and CaMKII were elevated, activating their respective signaling pathways. In contrast, the phosphorylation level of AMPK, a regulator of physiological cardiac remodeling, was reduced, suppressing its signaling pathway. This imbalance in phosphorylation levels ultimately led to cardiac remodeling and functional disorders [144].

Ubiquitination is a key PTM that regulates protein degradation, activity, and metabolic processes, thereby playing a central role in fundamental cellular processes including proliferation, apoptosis, and DNA repair [145], [146]. NASA exploration revealed that microgravity caused myocardial atrophy and functional decline in Drosophila, accompanied by myofibrillar remodeling and decreased cardiac output [147]. RNA sequencing revealed downregulation of sarcomere/ECM-related genes with significant upregulation of proteasomal genes, indicating that prolonged microgravity disrupted proteostasis, causing substantial alterations in protein turnover and misfolding [148], [149]. This suggests that proteostatic stress may represent a fundamental myocardial response to microgravity, with proteasome upregulation potentially underlying the mechanism of myocardial atrophy [147]. E3 ubiquitin ligases such as WW domain-containing E3 ubiquitin protein ligase 1 (WWP1) activate cardiac remodeling pathways through atypical ubiquitination [stabilizing rather than degrading dishevelled segment polarity protein 2 (DVL2) protein], while another E3 ubiquitin ligases Smurf1-mediated RUNX2 ubiquitination degradation, contributing to microgravity-induced bone loss [55], [94]. These findings reveal the dual regulatory mechanisms of ubiquitin-proteasome system in gravity-altered tissue remodeling: functioning through both classical degradation pathways (e.g., RUNX2) and nondegradative ubiquitination signaling (e.g., DVL2).

Acetylation and lactylation are two distinct PTMs that append different “short acyl” groups to lysine residues [150]. Rising acetyl-CoA induces histone acetylation that drives the neonatal cardiomyocyte cell cycle, whereas lactate accumulation installs lysine lactylation on histones with kinetics distinct from acetylation, thereby tuning transcriptional programs [151]. Under simulated microgravity conditions, cardiac protein lactylation shows an increasing trend in mice, while hepatic protein lactylation exhibits a decreasing trend, suggesting that metabolic changes induced by simulated microgravity and lactylation modifications display tissue-specific regulation [152], [153]. This observation aligns with NASA’s findings of distinct metabolic responses in various tissues of mice during actual spaceflight [53].

The aforementioned findings show that the PTMs play an indispensable role in the precise regulation of microgravity signals. Identification and discovery of more modification forms and key signaling molecules can provide newer and more effective targets for the precise prevention of microgravity-induced physiological effects.

## Systematic response characteristics of the body to microgravity

### Response characteristics of various tissues and organs in a microgravity state

**Fig. 5:**
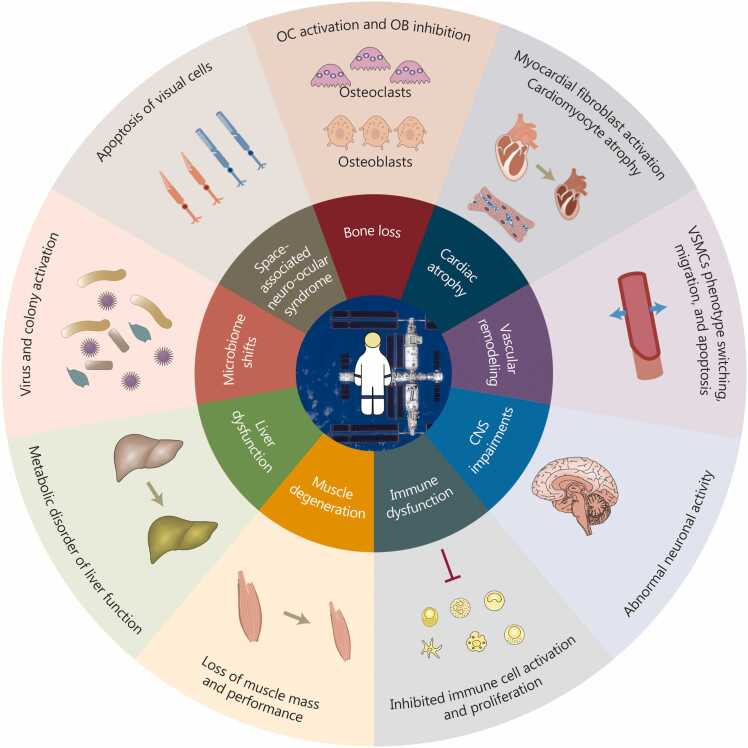
**Characteristics of changes in different tissues and organs under microgravity.** In microgravity environments, tissues and organs exhibit unique response characteristics, including bone loss and cardiac atrophy, vascular remodeling, immune dysfunction, CNS impairments, space-associated neuro-ocular syndrome, muscle degeneration, liver dysfunction, and microbiome shifts. VSMCs. Vascular smooth muscle cells; CNS. Central nervous system.

Distinct tissues and organs exhibit unique response characteristics under microgravity conditions. Microgravity induces a comprehensive shift in human physiology [154] (Fig. 5). Spaceflight leads to bone loss in load-bearing bones, with approximately 1.0%–1.5% monthly reduction in bone mineral density [76]. The cardiovascular system undergoes deconditioning, reducing orthostatic tolerance and triggering arterial remodeling due to altered fluid pressures [155]. Immune function is impaired as microgravity suppresses lymphocyte activity [156]. Furthermore, prolonged spaceflight causes structural changes in the brain, including enlarged ventricles and cerebellar displacement, and alters ocular structure, affecting vision [157], [158], [159], [160]. Astronauts experience metabolic shifts in liver function, characterized by elevated total cholesterol and low-density lipoprotein cholesterol, accompanied by reduced [53]. Also, significant alterations occur in gut microbiota composition and metabolites, marked by reduced diversity, depletion of beneficial bacteria (e.g., Firmicutes phylum and *Lactobacillus genus*), increased Bacteroidetes phylum and *Bacteroides genus*, along with notable changes in antibiotic resistance and virulence genes [161], [162].

The cellular response characteristics under microgravity conditions are closely related to their cytological features, with different cell types exhibiting distinct response patterns. For instance, under both real and clinostat-simulated microgravity conditions, the differentiation of pre-osteoblasts into mature osteoblasts is significantly impaired, accompanied by reduced proliferation, diminished differentiation capacity, and decreased osteogenic activity. Concurrently, osteoclast-mediated bone resorption is markedly enhanced, creating an imbalance in bone remodeling that drives weightlessness-induced bone loss [27], [55].

In summary, microgravity elicits tissue- and cell-specific responses, and in-depth investigation of these response characteristics is crucial for understanding the underlying mechanisms and developing effective countermeasures.

### Space aging syndrome

The physiological abnormalities exhibited by the human body during adaptation to space microgravity demonstrate all the characteristics of accelerated aging, with two remarkable similarities in their effects (Table 2) [53], [76], [155], [156], [157], [158], [159], [160], [163], [164], [165], [166], [167], [168], [169], [170], [171], [172], [173], [174], [175]. Both aging and exposure to space environment induce not just a decline in isolated systems, but rather a multisystem deterioration occurring at varying rates across nearly all physiological systems [176]. Spaceflight-induced physiological declines closely mirror cardiovascular aging [177]. After 6 months of spaceflight, astronauts exhibit 17%–30% increased carotid artery stiffness, equivalent to 10–20 years of terrestrial aging [178]. This accelerated vascular aging is likely driven by fluid shifts, elevated oxidative stress markers (superoxide dismutase, MMP2), and systemic insulin resistance [179]. Increased arterial stiffness elevates risks for cardiac events, stroke, and Alzheimer’s disease. Additionally, exposure to microgravity accelerates aging in the reproductive system. Male mice in low Earth orbit and rats in simulated microgravity conditions both demonstrate testicular tubule degeneration and a decrease in epididymal sperm counts. Concurrently, microgravity disrupts estrous cycles in female rodents and impairs in vitro follicular development [169], [171].

**Table 2:** Characteristics of physiological system changes in spaceflight-induced and natural aging.

**System**	**Space aging**	**Aging**	**Reference**
Musculoskeletal system	Rapid muscle atrophy and bone loss	Weakened muscle strength and decreased bone density	[76]
Cardiovascular system	Myocardial atrophy; Abnormal vascular function; Arrhythmia	Elevated blood pressure, arteriosclerosis, and weakened Myocardial function; Decreased cardiac function; Angiosclerosis	[155]
Nervous system	Changes in brain structure; Decreased memory; Cognitive impairment	Brain atrophy; Decreased memory; Slow reaction speed	[157], [158], [159], [160]
Immune system	Decreased resistance; Easy to infect; Cellular immune function impaired	Weakened immune response; Easy to infect; Increased tumor risk	[156]
Genetic repair ability	Decreased DNA repair ability; Accumulation of mutations	Decreased DNA repair speed; Accumulation of mutations	[164], [166], [167]
Psychological and cognitive influences	Sleep disorders; Depressed; Mood swing	Decreased memory; Decreased thinking ability; Decreased emotional stability	[163], [172]
Biological rhythm	Circadian rhythm disorder	Partial shortened rhythm cycle	[168], [173]
Hematopoietic stem cells	Decreased differentiation ability	Reduced efficacy and slower update speed	[156], [170]
Reproductive capacity	Testicular tubular degeneration in mice; Reduced epididymal sperm count; In vitro follicular development is impaired	Reproductive cell aging; Decreased reproductive ability; Decreased secretion of sex hormones	[169], [171]
Body glucose and lipid metabolism	Elevated low-density lipoprotein cholesterol; High-density lipoprotein cholesterol reduction; Elevated blood sugar levels and decreased insulin sensitivity	Abnormal glucose and lipid metabolism, elevated blood glucose levels; Hyperlipidemia; Diabetes	[53]
Risk of aging-related diseases	Increase	Increase	[165], [174], [175]

Space aging syndrome is linked to telomere dynamics, DNA damage, and oxidative stress [164], [166], [167]. Telomeres, chromosomal end-caps that prevent genetic instability, typically shorten with aging and environmental stress [180]. NASA studies found that astronauts’ telomeres elongated during space missions (6–12 months), but rapidly shortened upon return to Earth, accompanied by increased chromosomal inversions and genomic instability [181], [182]. Persistent oxidative stress in space disrupts telomerase activity and directly damages telomeres, accelerating cellular aging and elevating cancer risk [161], [182]. Spaceflight-induced persistent oxidative stress not only disrupts telomerase activity, leading to telomere dysfunction and aging, but also directly causes oxidative damage to telomeres [183], [184]. In aerospace and simulated space environments, a pro-oxidative state (elevated oxidative enzymes and decreased antioxidant enzymes) has been observed in multiple organs and cells [185]. Excessive ROS reacts with lipids, proteins, and DNA, leading to aging, diseases, and cell death [186], [187], [188]. Furthermore, numerous studies have found that astronauts experience persistent mitochondrial stress during spaceflight [182], [189], [190], with mitochondria being recognized as the most significant cellular source of ROS. Consequently, microgravity-induced oxidative stress can accelerate aging in various astronaut tissues and organs, including the brain, muscles, bones, and others.

### Changes in secretory factors under microgravity conditions

Secretory factors are now redefined as ubiquitous chemical messengers for intercellular communication, encompassing cytokines, neurotransmitters, neuropeptides, noncoding RNAs, metabolic molecules, extracellular vesicles (EVs), and others [10], [191], [192]. Their functions include participation in organismal metabolism, coordination of organ and systemic activities, and maintenance of homeostasis [193]. These factors undergo significant changes during spaceflight.

#### Exosomes

Significant changes occur in plasma exosomes, exhibiting quantitative alterations and differences in surface markers in the human body during spaceflight. NASA’s twin study revealed a 30-fold increase in the number of plasma exosomes of astronauts compared with ground controls, despite having similar sizes [161]. Plasma exosomes from astronauts were enriched with the proinflammatory monocyte marker CD14 and contained detectable levels of basigin and integrin β1-proteins associated with cancer progression and inflammation, neither of which was present in control exosomes [194].

#### MiRNA

The levels of miRNA in plasma also change during spaceflight. In 2020, NASA conducted screening analyses and obtained datasets of circulating miRNAs sensitive to microgravity and radiation. Among these, miR-125, miR-16, and let-7a were confirmed to regulate vascular damage induced by simulated deep-space radiation [195]. Additionally, human bed-rest studies simulating microgravity demonstrated significant alterations in circulating miRNA profiles of serum, which were correlated with the degree of bone loss [196].

#### Cytokine

As intercellular signaling molecules, cytokines play critical regulatory roles in physiological processes such as immunity, inflammation, hematopoiesis, cell growth, and repair. Widespread expression changes in plasma cytokines have been observed during actual spaceflight. NASA data from long-duration ISS missions indicate a significant increase in pro-inflammatory cytokines (e.g., IL-1, IL-6, IL-10, C-C motif chemokine ligand 2, and tumor necrosis factor-α) in astronaut serum, while no significant changes were detected in cytokines secreted by regulatory T cells (e.g., IL-4, IL-5). These findings suggest the occurrence of tissue damage and inflammation in the body. Concurrently, the significant increase in growth factors and hematopoietic factors (e.g., vascular endothelial growth factor, thrombopoietin, granulocyte-macrophage colony-stimulating factor) reflects ongoing tissue repair processes under stress conditions [197], [198].

#### Metabolites

Metabolic changes in urine or blood samples can be used to assess the metabolic status of astronauts, with the most pronounced alterations observed in metabolites related to bone metabolism [199]. For instance, in multiple actual spaceflight missions, significant increases in bone resorption marker (C-terminal telopeptide of type I collagen) and decreases in bone formation markers (type I N-terminal propeptide, type I C-terminal propeptide, alkaline phosphatase, and osteocalcin) have been observed during flight, along with elevated urinary calcium concentrations. These changes collectively reflect substantial alterations in bone metabolism [8].

### Interaction between tissues and organs under microgravity

**Fig. 6:**
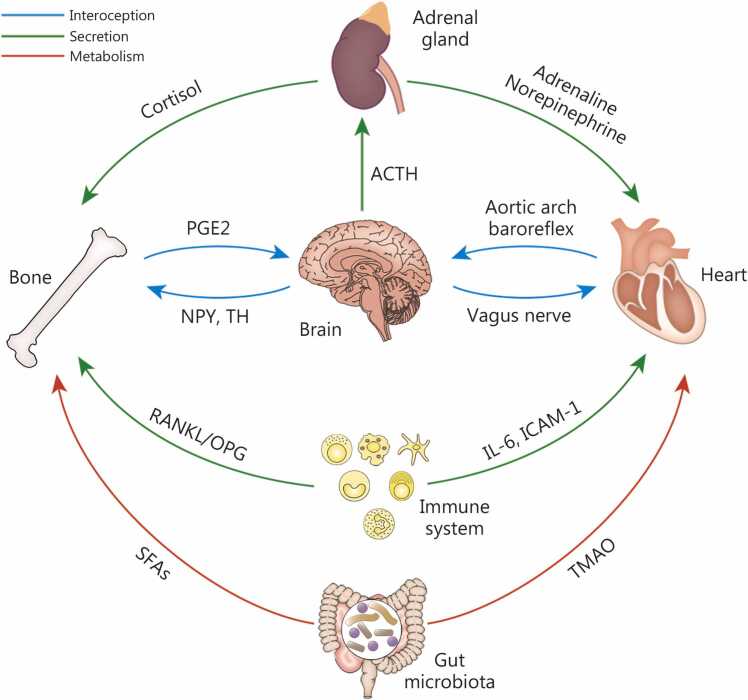
**Interaction between tissues and organs under weightlessness conditions.** In microgravity environments, tissues and organs (such as Bone, brain, heart, adrenal gland, immune system, and gut microbiota) exhibit complex adaptive or pathological changes through interactions among interoceptive, neuroendocrine, immune, and metabolic systems. ACTH. Acetylthiocholine; PGE2. Prostaglandin E2; NPY. Neuropeptide Y; TH. Tyrosine hydroxylase; RANKL. Receptor activator of nuclear factor κB ligand; OPG. Osteoprotegerin; IL-6. Interleukin-6; ICAM-1. Intercellular adhesion molecule-1; SFAs. Saturated fatty acids; TMAO. Trimethylamine N-oxide.

The interaction between tissues and organs in a weightless environment is a multisystem coordinated regulatory process, involving dynamic equilibrium at molecular, cellular, and organ levels [8], [200]. In this environment, tissues and organs exhibit complex adaptive or pathological changes through the interplay of interoception, neuroendocrine, immunoregulation, and metabolic systems (Fig. 6). The underlying mechanisms involve multi-organ coordination and dysregulation of signaling pathway homeostasis.

#### Interoception

Interoception is the process mediating brain-periphery interaction, by which metabolic responses to stimuli are relayed to the central nervous system (CNS), which then integrates and returns signals to peripheral organs to maintain homeostasis [201], [202]. Prostaglandin E2 level in bones decreases under microgravity conditions, stimulating the expression of neuropeptide Y (NPY) and tyrosine hydroxylase (TH) in the hypothalamus through skeletal interoception [203]. These changes in TH levels elevate sympathetic nerve tension, inducing bone resorption and white adipose tissue lipolysis. However, increased NPY levels alter energy expenditure and metabolism, thereby maintaining the balance between bone formation and resorption [203]. Without gravity, the functional characteristics of the muscular layers in hollow digestive organs (such as the stomach and intestines) undergo alterations, maintaining adaptive regulation of digestive function through proprioceptive feedback mechanisms [204]. Interoception regulates the CNS control over digestive activities by perceiving signals such as gastrointestinal mechanical stretching and hormone secretion, thereby modulating vagal afferent pathways [205]. Microgravity-induced fluid redistribution changes cardiac interoceptive sensitivity. The interoceptive system regulates autonomic nervous system functions (including heart rate and vascular tone) by perceiving signals such as heart rate and blood pressure fluctuations, thereby activating the vagus nerve-brainstem-hypothalamus pathway to maintain hemodynamic stability [206].

#### Neuroendocrine

Microgravity disrupts the balance of inter-tissue regulatory networks by altering the production, transmission, and reception efficiency of neuroendocrine signaling molecules [203], [207]. The human stress response activates the hypothalamic-pituitary-adrenal axis. The hypothalamus secretes corticotropin-releasing hormone, which acts on the anterior pituitary to stimulate adrenocorticotropic hormone (ACTH) secretion [208], [209]. Upon entering systemic circulation, ACTH stimulates the adrenal cortex to secrete glucocorticoids such as cortisol [209]. Cortisol subsequently influences multiple physiological processes, including metabolism, immune function, and cardiovascular regulation [210]. The body fluid redistribution occurs in a weightless environment, significantly impacting the cardiovascular system [211]. Consequently, the body activates the sympathetic nervous system (SNS) to maintain blood pressure stability. Specifically, SNS excitation stimulates the adrenal medulla to release epinephrine and norepinephrine. As a result, these hormones increase heart rate and enhance myocardial contractility to boost cardiac output, while simultaneously inducing vasoconstriction to elevate peripheral resistance and sustain blood pressure. However, chronic SNS hyperactivity and prolonged elevation of epinephrine/norepinephrine secretion may exert detrimental cardiovascular effects [212]. This persistent stimulation can lead to pathological consequences, including myocardial hypertrophy and vascular stiffness. These maladaptive changes mirror some cardiovascular deconditioning phenomena observed in long-duration spaceflight [213].

#### Immunoregulation

Microgravity disrupts immune function by impairing the differentiation, activation, and metabolic processes of immune cells. For instance, T cell activation is suppressed, monocytes exhibit enhanced proinflammatory states (with dysregulated secretion of cytokines such as IL-6), and at the same time, the antigen presentation capacity of dendritic cells is diminished. As a result, the efficiency of adaptive immune responses is reduced [214], [215]. Furthermore, this immune dysregulation may influence distant organs through the cytokine network. Abnormal interactions between endothelial cells and immune cells altered intercellular adhesion molecule-1 expression may accelerate the progression of atherosclerosis [216]. Immune cells actively modulate the critical balance between osteoblast-mediated bone formation and osteoclast-mediated bone resorption. Conversely, the skeletal system plays a fundamental role in immune cell differentiation through its hematopoietic stem cell niche [217]. Osteoblasts play a key role in hematopoietic stem cell (HSC) regulation by producing essential niche factors such as osteopontin and activating multiple signaling pathways, including neurogenic locus notch homolog protein/Jagged canonical notch ligand (Notch/Jagged), angiopoietin-1/tyrosine kinase with immunoglobulin-like and EGF-like domains 2 (Ang-1/Tie2), and Wnt/β-catenin cascades. This regulation helps maintain the balance between HSC maintenance and immune cell lineage commitment [218].

#### Metabolic system

In the weightlessness environment, the metabolic system undergoes significant alterations through complex inter-tissue interaction mechanisms, involving multiple levels such as energy metabolism reprogramming, EV-mediated signaling, and dysregulation of core organ functions [161], [219]. Adipose tissue plays a pivotal role in systemic metabolic regulation, exhibiting endocrine dysfunction under microgravity [220], [221], and secretes EVs and adipokines (e.g., angiopoietin-like 2) that modulate metabolic homeostasis via inter-tissue communication [222]. These EVs carry miRNAs and proteins that remotely regulate hepatic glucose metabolism and bone mechanical responses, but this cross-tissue signal transduction becomes disrupted under microgravity [223]. Meanwhile, impairment of the adipose-nervous system’s leptin signaling pathway leads to dysregulation of energy homeostasis, which may exacerbate microgravity-induced insulin resistance [224]. Additionally, microgravity disrupts the bone-muscle-fat metabolic axis by inhibiting the Wnt/β-catenin and Hippo signaling pathways, which suppress osteogenic differentiation. This suppression results in a bidirectional metabolic imbalance: bone formation decreases while lipid deposition increases. Meanwhile, decreased osteocalcin secretion from bone tissue weakens its regulatory capacity on adipose tissue thermogenesis, while muscle atrophy-induced aberrant myokine (e.g., irisin) secretion forms a vicious cycle in bone-muscle-fat crosstalk [214], [225]. Moreover, the liver’s pivotal metabolic function undergoes remodeling. As the core metabolic organ, the liver exhibits significant metabolic reprogramming under microgravity conditions. Serving as the hub of iron metabolism, aberrant elevation of hepatic hepcidin expression induces iron overload, subsequently triggering bone loss. The iron chelator deferoxamine can inhibit iron deposition and mitigate bone loss [226]. The metabolic disorders caused by microgravity are also reflected in the intestine. Alterations in gut microbiota and their metabolic products (such as trimethylamine N-oxide) induced by spaceflight or simulated microgravity are closely associated with changes in cardiovascular function [227].

The metabolic regulation of the organism in a weightless environment is a complex, multisystem interaction process. A deep understanding of these interactive regulatory mechanisms is of great significance for developing effective protective measures against physiological dysfunction. Future studies should explore the intricate relationships between these systems and provide important theoretical foundations for long-term space exploration and related medical studies.

## Accurate monitoring and protection

Recent studies have gradually revealed the holistic and systemic characteristics of stress-induced damage caused by the extreme space environment [165], [228]. Therefore, considering the integral and systematic patterns of functional changes in various tissues and organs induced by space microgravity, as well as the multilevel gravitational response characteristics, more precise molecular-level early warning monitoring methods and protective countermeasures should be developed. This may enable real-time dynamic monitoring and robust protection of human health in space environments.

### Establishment and development of new monitoring methods

Current space health monitoring during spaceflight is inadequate, as it relies heavily on pre- and post-flight ground-based tests [178]. It faces limitations in detection indicators, sensitivity, and specificity, and research for long-duration missions [229]. Therefore, space agencies have been committed to establishing new types of real-time and sensitive in-orbit health monitoring systems.

#### Targeting sensitive biomarkers

MiRNAs offer significant advantages as non-invasive liquid biopsy biomarkers for aerospace medicine, enabling early warning of microgravity- and radiation-induced health risks [230]. They mediate and indicate microgravity-induced vascular remodeling, immune dysfunction, and bone loss [231], thus serving as effective targets for rapid in-flight monitoring.

Surface-enhanced raman scattering (SERS) based on surface plasmon resonance (SPR) has demonstrated exceptional advantages in recent years beyond ultra-high sensitivity, including strong environmental adaptability, minimal sample preprocessing requirements, capability for rapid multiparameter analysis, and high potential for miniaturization and integration [232], [233]. This enables rapid, sensitive, specific, and multiplexed detection of the physiological indicators of astronauts under extreme conditions, particularly during spaceflight. Building upon SPR-SERS, current investigations focus on developing portable, multiplexed, and reproducible SERS chips targeting specific biomarkers such as miRNAs and small biochemical molecules, aiming to achieve real-time in-orbit health monitoring for astronauts [234].

#### In-orbit noninvasive health evaluation technology based on advanced imaging technology

Two-photon imaging technology is a novel imaging technique developed based on the two-photon absorption/excitation phenomenon, demonstrating unique advantages in the field of aerospace medicine [235]. This technology enables *in vivo* noninvasive microscopic imaging of the structure, composition, and function of superficial skin cells, overcoming the limitations of traditional methods that cannot achieve real-time monitoring at the cellular scale. Ground-based studies have shown that using two-photon microscopy allows real-time monitoring of superficial skin structure and composition in humans during head-down bed rest to simulate microgravity, capturing changes in human oxidative stress status [236], [237]. After design optimization, the two-photon microscope was transported to the Tiangong Space Station through the Tianzhou-15 cargo spacecraft, reporting for the first time information about the in-orbit cellular structure and metabolic components of astronauts through skin imaging. It clearly presented the three-dimensional distribution of the skin structure and cells of astronauts with sub-micron resolution [238]. This technology enables the detection of stress-induced changes in mitochondrial metabolic function caused by spaceflight, thereby providing new tools and methods for aerospace medicine research on the in-orbit, cellular-level health monitoring of astronauts.

#### Handheld MyotonPRO device for measuring the changes in muscle hardness

The handheld MyotonPRO device noninvasively assesses muscle health status in microgravity environments by measuring the changes in muscle stiffness [239]. Its measurement principle is based on the algorithmic calculations of damped natural oscillation reflection signals [239]. Study has validated strong consistency between myoton technology and shear wave elastography, thus demonstrating reliability and utility across clinical research, healthy populations (including both sexes), and short-term microgravity conditions during parabolic flights [240]. Research findings indicate a direct correlation of muscle tissue stiffness with the effectiveness of countermeasures during space missions exceeding 180 d [241].

#### Distortion product otoacoustic emission monitoring of intracranial pressure changes

Distortion product otoacoustic emissions (DPOAEs) are nonlinear cochlear signals composed of distortion and reflection components, with the former linked to active amplification and the latter to basilar membrane mechanics [242]. The stimulus frequency ratio (f2/f1) significantly influences overall DPOAE levels, although its specific effects on individual components require further investigation. High-frequency DPOAEs (e.g., in extended high-frequency ranges of 9–16 kHz) exhibit greater sensitivity to age-related changes, potentially reflecting cochlear aging or early damage [243]. The application of DPOAEs as a noninvasive technique for monitoring intracranial pressure changes can help evaluate vision-related risks in microgravity environments. This approach has undergone feasibility studies aboard the ISS [244].

#### Other in-orbit health monitoring technologies

Current space health monitoring has developed into a multi-organ system approach. The cardiovascular system is monitored through continuous tracking of astronauts’ vital signs via wearable devices such as smartwatches, combined with handheld ultrasound imaging for organ evaluation [245]. The skeletal system is assessed using novel piezoelectric-based sensors that enable real-time monitoring of bone health while also promoting bone tissue repair [246]. The nervous system is evaluated by measuring the impact of the space environment on the CNS through neurovestibular change indices and brain function monitoring [200]. The immune system is profiled via multi-omics molecular analysis techniques, allowing for the monitoring of immune function changes under microgravity conditions [247].

### Research and development of effective adversarial protection methods based on targets

Astronauts face multiple health risks during long-duration space missions, including cardiovascular dysfunction, skeletal muscle atrophy, bone loss, and comprehensive physiological effects such as circadian rhythm disruption and immune-endocrine disorders. Current protective measures mainly include active exercise, equipment-assisted protection, and pharmacological interventions. However, data from the ISS indicate that, even with existing protective measures partially alleviating symptoms, astronauts still experience up to 20% muscle mass loss, persistent bone loss, as well as cardiovascular structural and metabolic abnormalities [177]. Therefore, more targeted medical protection strategies and intervention measures need to be urgently developed based on existing protective approaches. Space medical protection strategies based on biomolecular targets have gained increasing attention in recent years. Establishing comprehensive protection and intervention strategies for organisms under space microgravity conditions by identifying key targets for whole-body and crucial organ damage is of great significance for ensuring the successful implementation of national major human spaceflight projects [248], [249].

#### Small nucleic acid drugs

Small nucleic acid drugs, including antisense oligonucleotides, small interfering RNAs (siRNAs), and miRNAs, have emerged as research hotspots in clinical targeted therapy and precision medicine due to their advantages of high specificity, convenient design, short development cycles, and abundant target options [250]. MiRNA-214 is highly expressed in the serum and bone tissue of simulated microgravity model mice [115]. Preliminary studies have confirmed the dual regulatory role of miR-214 in both bone formation and bone resorption [115], [251], [252]. The antisense oligonucleotide of miR-214 has demonstrated excellent therapeutic effects against microgravity-induced bone loss, osteonecrosis, and osteoarthritis, while simultaneously counteracting vascular remodeling [195]. This makes it an effective protective measure against skeletal and vascular remodeling caused by microgravity.

#### Extracellular vesicle-based drugs

Exosomes are key mediators of intercellular communication, stably transporting bioactive factors, including proteins, RNA, and DNA, to exert biological functions and interactions among different cell types [253], [254], [255]. Due to their low immunogenicity, high biocompatibility, and ability to carry diverse biomacromolecules, they serve as promising targeted delivery platforms, therapeutic agents, and intervention targets for developing effective adversarial protection strategies [256]. Given their significant role in regulating bone metabolism, one study has used erythropoietin-producing hepatocellular receptor A2-targeted exosomes to modulate miR-214 transport between osteoclasts and osteoblasts, thus achieving bidirectional regulation of both bone formation and bone resorption processes [257]. Reparative M2-like macrophage-derived exosomes (M2-Exos) restore bone remodeling balance at the cellular level by promoting osteoblast differentiation and inhibiting osteoclast differentiation, thereby exerting bone-protective effects [258]. METTL3-modified exosomes (METTL3-EXO) enhance osteogenic differentiation and suppress adipogenic differentiation, alleviating bone loss [259]. This approach provides a novel therapeutic strategy for weightlessness-induced bone loss.

#### Natural small molecule compounds

Natural small-molecule compounds have demonstrated unique advantages in counteracting multisystem pathological changes induced by spaceflight weightlessness. Studies have indicated that Panax quinquefolius saponins from the stems and leaves of American ginseng can not only alleviate simulated weightlessness-induced bone loss and cardiomyocyte remodeling but also promote long-bone vascular regeneration, thus achieving comprehensive protection against weightlessness-induced osteopenia and cardiovascular dysfunction [260], [261]. Ginseng stem-leaf saponins (DS) effectively counteract spaceflight depression caused by simulated weightlessness, thereby relieving tail suspension-induced depression and spatial memory decline in mice [262]. Additionally, a formula displays remarkable therapeutic effects against weightlessness-induced muscle atrophy [263]. Developing systematic countermeasure strategies based on traditional Chinese medicine to address physiological pathologies caused by weightlessness represents one of the future directions for investigations on microgravity countermeasures.

## Conclusions

Microgravity, as the most distinctive environmental factor during spaceflight, elicits multi-level responses in living organisms, spanning from molecular to systemic scales. This review systematically elaborates on the hierarchical response mechanisms of biological systems to microgravity and proposes an integrated signaling cascade framework, from structural sensing to calcium signal transduction, epigenetic and post-translational regulation, to inter-tissue crosstalk, offering a novel perspective for understanding the molecular basis of spaceflight-induced pathophysiology.

Moreover, the space environment is inherently complex, encompassing multiple factors such as microgravity, radiation, hypomagnetic fields, confined spaces, shifts in the microecology, as well as noise and vibration. Therefore, studying physiological changes under actual spaceflight conditions requires a comprehensive consideration of these combined effects. With the advancement of biotechnologies and their deep integration with computational science, electronics, nanomaterials, and artificial intelligence, the development of real-time precision monitoring and targeted protective technologies will further support the implementation of human spaceflight programs.

## Abbreviations

**Table:** 

ACTH	Adrenocorticotropic hormone
AMPK	AMP-activated protein kinase
Ang-1	Angiopoietin-1
Ano1	Calcium-activated chloride channel
ATF4	Activating transcription factor 4
ATP	Adenosine triphosphate
Bcl-2	B-cell lymphoma 2
BMP	Bone morphogenetic protein
BMSCs	Bone marrow mesenchymal stem cells
*C. elegans*	Caenorhabditis elegans
CaM	Calmodulin
CaMKII	Calmodulin-dependent protein kinase II
CaMKK2	Ca²⁺/Calmodulin-dependent protein kinase kinase 2
CKIP-1	Casein kinase 2 interacting protein-1
CNS	Central nervous system
DPOAEs	Distortion product otoacoustic emissions
DVL2	Dishevelled segment polarity protein 2
ECM	Extracellular matrix
Ep300	E1A binding protein p300
ERK	Extracellular regulated protein kinases
EVs	Extracellular vesicles
H3K4me3	Histone H3 lysine 4 trimethylation
Hax1	HCLS1-associated protein X-1
HDAC4	Histone deacetylase 4
HSC	Hematopoietic stem cell
HUVEC	Human umbilical vein endothelial cell
IL-6	Interleukin-6
iPSC	Induced pluripotent stem cell
IQGAP1	IQ motif-containing GTPase-activating protein 1
ISS	International Space Station
LINC	Linker of nucleoskeleton and cytoskeleton
lncRNA	Long noncoding RNA
miRNA	MicroRNA
Neat1	Nuclear paraspeckle assembly transcript 1
NPY	Neuropeptide Y
PIEZO1	Piezo type mechanosensitive ion channel component 1
PTMs	Post-translational modifications
ROS	Reactive oxygen species
RUNX2	Runt-related transcription factor 2
RyR	Ryanodine receptor
SERCA	Sarco/endoplasmic-reticulum Ca²⁺-ATPase
SERS	Surface-enhanced raman scattering
Setdb1	SET domain bifurcated histone lysine methyltransferase 1
Smurf1	SMAD-specific E3 ubiquitin protein ligase 1
SNS	Sympathetic nervous system
TAZ	Transcriptional co-activator with PDZ-binding motif
TH	Tyrosine hydroxylase
TRP	Transient receptor potential
TRPC	Transient receptor potential canonical
TRPV4	Transient receptor potential vanilloid 4

## Ethics approval and consent to participate

Not applicable.

## Funding

This work was supported in part by the National Natural Science Foundation of China (82192882, 32200972), the National Key Research and Development Project (2022YFA1104203i), the Postdoctoral Fellowship Program of CPSF (GZC20233414), and the China Postdoctoral Science Foundation (2024T171124, 2024M764151).

## CRediT authorship contribution statement

WJS, RKD, YHL, GHZ, JWL, SKL, and YXL conceived and designed the whole project. WJS wrote the manuscript, RKD drew the pictures. All the authors discussed, revised, and approved the manuscript.

## Data Availability

Not applicable.
